# Efficient and Non-Toxic Biological Response Carrier Delivering TNF-α shRNA for Gene Silencing in a Murine Model of Rheumatoid Arthritis

**DOI:** 10.3389/fimmu.2016.00305

**Published:** 2016-08-19

**Authors:** Jialin Song, Yinghui Chen, Shichao Jiang, Kejia Yang, Xiaoming Li, Xiaotian Zhao, Yuanming Ouyang, Cunyi Fan, Weien Yuan

**Affiliations:** ^1^Shanghai Jiao Tong University Affiliated Sixth People’s Hospital, Shanghai, China; ^2^Department of Neurology, Jinshan Hospital, Fudan University, Shanghai, China; ^3^Department of Orthopedics, Shandong Provincial Hospital Affiliated to Shandong University, Jinan, Shandong, China; ^4^School of Pharmacy, Shanghai JiaoTong University, Shanghai, China; ^5^Shanghai Sixth People’s Hospital East Campus, Shanghai University of Medicine and Health, Shanghai, China

**Keywords:** rheumatoid arthritis, RNA interference, TNF-α, PDAPEI

## Abstract

Small interfering RNA (siRNA) is an effective and specific method for silencing genes. However, an efficient and non-toxic carrier is needed to deliver the siRNA into the target cells. Tumor necrosis factor α (TNF-α) plays a central role in the occurrence and progression of rheumatoid arthritis (RA). In this study, we pre-synthetized a degradable cationic polymer (PDAPEI) from 2,6-pyridinedicarboxaldehyde and low-molecular-weight polyethyleneimine (PEI, Mw = 1.8 kDa) as a gene vector for the delivery of TNF-α shRNA. The PDAPEI/pDNA complex showed a suitable particle size and stable zeta potential for transfection. *In vitro* study of the PDAPEI/pDNA complex revealed a lower cytotoxicity and higher transfection efficiency when transfecting TNF-α shRNA to macrophages by significantly down-regulating the expression of TNF-α. Moreover, the complex was extremely efficient in decreasing the severity of arthritis in mice with collagen-induced arthritis. PDAPEI delivered TNF-α shRNA has great potential in the treatment of RA.

## Introduction

Rheumatoid arthritis (RA) is a chronic systemic autoimmune disorder with complex causes, which leads to cartilage loss and synovial inflammation (1, 2). Many different immune cells and the cytokines they produce contribute as mediators to RA pathogenesis in cartilage and bone tissue (3, 4). Among the cytokines produced, TNF-α is the key mediator of inflammation in RA, and macrophages are highly activated cells in the inflamed synovial membranes of RA (5, 6). Antagonists of biotherapies can neutralize TNF-α at the protein level and decrease joint inflammation, but to date, no long-term studies have been fully conducted (7, 8).

Anti-TNF-α antibodies has shown its efficacy in RA and is widely used clinically (9, 10). However, lower mRNA concentration regulated by gene transcription shows a more efficient and long-lasting way (11). Small interfering RNA (siRNA) silencing blocks the expression of specific genes and has great potential for treating a wide range of illnesses (12–14). Much work has already been done on the silencing of TNF-α and on the downregulation of both local and systemic inflammation in the treatment of RA (15–17). However, siRNA-based therapies for RA have yet to overcome the obstacles of designing vehicles capable of both protecting and delivering siRNAs (18). Viral and non-viral vectors have been widely used for gene delivery and delivering an adequate amount of siRNA to target cells (19, 20). Cationic polymers have the advantage of better biocompatibility, lower immunogenicity, and easier modification, which can form polyplexed structures with negatively charged siRNA (21, 22). Polyethylenimine (PEI) has also been widely used in gene delivery, but its cytotoxicity limits its further application (23). Low-molecular-weight PEI (<20 KDa) cross-linked by degradable linkers, however, shows a lower cytotoxicity and higher transfection efficiency (24, 25). In the present study, PEI 1.8 kDa was cross-linked by 2,6-pyridinedicarboxaldehyde (PDA) to develop a biodegradable low-molecular-weight cationic polymers (PDAPEI). In an acidic environment, the PDAPEI shows self-degradation into non-toxic low-molecular-weight PEI and 2,6-PDA (26).

Herein, we investigated TNF-α shRNA delivered by PDAPEI for the treatment of RA. We examined its characteristics, cytotoxicity, transfection efficiency, and gene expression on macrophages. PDAPEI/pDNA was also used to treat mice with collagen-induced arthritis (CIA), and the therapeutic efficacy was evaluated using histological examination.

## Materials and Methods

### Materials

Polyethylenimine 1.8 kDa and 25 kDa and anhydrous ethylene dichloride were purchased from Sigma-Aldrich. 2,6-PDA was purchased from TCI (Shanghai) Development Co., Ltd. Cellulose membranes (MWCO 10,000 Da) were purchased from Thermo Scientific. Poly (ethylene glycol) (PEG) standards kit (ranging from 106 to 20,100 Da in molecular weight) was purchased from Polymer Standards Service GmbH. Water was purified using a milli-Q instrument (Millipore). All the reagents were used without further purification. For TNF-α gene silencing, Plasmid DNA encoding green fluorescence protein (GFP) and mouse TNF-α shRNA were constructed by Bioroot Biology (Shanghai, China) according to a previous report that used the following sequence: 5′-GACAACCAACUAGUGGUGCdTdT-3′. The RAW 264.7 macrophage cell line was purchased from the cell bank of the Chinese Academy of Sciences (Shanghai, China).

### Synthesis of Polymers

First, 1 mmol PEI 1.8 kDa and 20 ml anhydrous ethylene dichloride solution were mixed until completely dissolved. Then, 2 mmol PDA in 20 ml anhydrous ethylene dichloride solution was added and stirred for 48 h at room temperature. After removing the solvent by evaporation, the residue was dissolved in deionized water and dialyzed through the cellulose membrane (<10 k). Then, following lyophilization, we were left with the yellow polymers, referred to as PDAPEI.

### Preparation and Characterization of PDAPEI/pDNA Complex

PDAPEI/pDNA complexes were prepared at different weight/weight (w/w) ratios of 1–50. First, PDAPEI was dissolved with deionized water (2 mg/ml) and pDNA (2 mg/ml). Then, the PDAPEI solution was diluted to different concentrations and added rapidly into the pDNA solution. Next, the solutions were incubated at room temperature for 30 min, and PEI/pDNA complexes and naked pDNA were simultaneously prepared as controls.

Agarose gel electrophoresis assay was used to evaluate the condensation ability of PDAPEI/pDNA complexes at different w/w. The particle size and zeta potential of PDAPEI/pDNA complexes were measured using a particle size analyzer (Brookhaven Instruments) and a zeta potential analyzer (Zetasizer Nano, Malvern Instruments) at different w/w ratios. Zeta potential across a range of pH (5.4, 6.4, 7.4, 8.4, 9.4) was measured to analysis its stability and degradation. The morphology of the PDAPEI/pDNA complexes was observed by Transmission electron microscopy (JEOL JEM 2010 system).

### *In Vitro* Study

#### Macrophage Culture

The RAW 264.7 macrophage cell line was cultured in Roswell Park Memorial Institute-1640 (RPMI-1640) containing 10% fetal bovine serum, 100 IU/ml penicillin, and 10 μg/ml streptomycin at 37°C inside a 5% CO2 atmosphere.

#### Cytotoxicity of the PDAPEI/pDNA Complex

For the cytotoxicity of the PDAPEI/pDNA complex in RAW 264.7, cells (1.0 × 10^4^/well) were seeded into 96-well plates and incubated for 24 h. Then, a 10-μl PDAPEI/pDNA solution (w/w from 1 to 50) was added into each well for an additional 4-h incubation. A cell counting Kit-8 (CCK-8) reagent was used to evaluate the cytotoxicity of the complexes. Briefly, 10 μl of the CCK8 reagent was added into each well and cultured for another 2 h and measured at 450 and 630 nm using multifunctional microplate reader (SpectraMax M3 Multi-Mode Microplate Reader). Similarly, PEI/pDNA complexes and PEI 1.8 kDa/pDNA were examined as controls.

#### Transfection Efficiency

The constructed shTNF-α was encoded with a GFP for determining the transfection efficiency. RAW 264.7 (5–10 × 10^4^/ml) were seeded into 12-well plates and cultured for 24 h. Next, the medium was replaced by solutions containing 200 μl of PDAPEI/pDNA complexes of different w/w ratios and 800 μl RPMI-1640 for an additional 4 h of incubation. Then, the transfection medium was removed, and 1 ml of RPMI-1640 (10% FBS and 1% antibiotics) was added for 48-h incubation. We also prepared PEI 1.8 kDa/pDNA as the negative control and PEI 25 kDa/pDNA (w/w ratio of 2) as the positive control. A fluorescent microscope (Olympus, Tokyo, Japan) and flow cytometer (BectoneDickinson, Franklin Lakes, NJ, USA) were used to quantify the GFP-positive cells.

#### TNF-α Silencing Experiment

To evaluate the gene silencing efficiency of PDAPEI/pDNA *in vitro*, RAW 264.7 cells were seeded in 12-well plates and cultured for 24 h. The cells were treated with PDAPEI/pDNA complexes (w/w ratio of 20) for 4 h, PEI 1.8 kDa/pDNA and PBS was used as a negative control and PEI 25 kDa/pDNA (w/w ratio of 2) was used as a positive control. Then, the transfection medium was replaced with fresh RPMI-1640 (10% FBS and 1% antibiotics) and incubated for 24/48 h. ELISA measurement was applied to detect TNF-α expression in the supernatant according to the manufacturer’s instructions (R&D Systems, Minneapolis, MN, USA). Total RNA was extracted from the cells using RNeasy^®^Mini Kit (Qiagen, Valencia, CA, USA) and reverse transcribed into cDNA using High-Capacity cDNA Reverse Transcription Kit (Applied Biosystems). The cDNAs for TNF-α were amplified by RT-PCR under the following conditions: denaturation at 95°C for 10 s; 40 cycles of amplification at 95°C for 15 s, and 60°C for 30 s. The specific primers used to detect mouse TNF-α (forward: 5′-AGTGGAGGAGCAGCTGGAGT-3′; reverse5′-TCCCAGCATCTTGTGTT TCTG-3′) were synthesized and purified by TAKARA, Inc. Then, the qPCR products were subjected to electrophoresis on 2% agarose gels and visualized with UV light.

### Animal Study

#### Animal Model and Treatment

Healthy male DBA/1 mice were purchased from the Institute of Zoology, Chinese Academy of Sciences (Beijing, China). All animals survived under specific pathogen-free (SPF) environment in a laboratory within the animal facility located at the school of pharmacy, Shanghai Jiao Tong University. All mice were housed under standardized laboratory conditions and monitored to observe changes in ordinary conditions and activities. Animal care and use were in accordance with the guidelines established by the Administration of Affair Concerning Laboratory Animals for Shanghai JiaoTong University, the National Institutes of Health Guide for care and Use of Laboratory Animals (GB14925-2010) and the Regulations for the Administration of Affairs Concerning Experimental Animals (China, 2014). The murine CIA model was prepared according to a previous study (27). Type II collagen was used as an autoantigen for first immunization and booster immunization (3 weeks after first immunization until the model was successfully made, mice without symptoms were eliminated). Twenty-four CIA mice were randomly divided into four groups (six mice per group): saline, PEI 1.8 kDa/DNA complex, PEI 25 kDa/pDNA complex and PDAPEI/pDNA complex, after receiving their booster immunization. Each group received intravenous injections of saline, pDNA (1.5 mg/kg shRNA), PEI 25 kDa/pDNA complex (at w/w ratio of 2, 1.5 mg/kg shRNA) and PDAPEI/pDNA complex (at proper w/w ratio, 1.5 mg/kg shRNA) according to the *in vitro* study results 1 week after booster immunization. The treat injection was repeated once a week until 5 weeks when the therapeutic efficacy was analyzed.

#### Clinical Examination

Arthritic severity was recorded based upon the clinical scores of all four paws. Clinical severity of arthritis for each paw was classified as follows: 0 (normal joints), 1 (slight redness and/or swelling), 2 (pronounced edematous swelling), and 3 (joint deformity and rigidity). The total clinical score was calculated by averaging all four paws scores. Hind paw edema was also measured by digital Vernier caliper to determine the severity.

#### Measurement of TNF-α in Knee

Before the mice were sacrificed for histological examination, synovial fluid was collected from the knees to measure TNF-α by ELISA and western blot, as per the instructions of the manufacturer and our previous study.

#### Histological Examination

At 6 weeks after the first immunization, the knee joint tissues of each group were collected and fixed in fixative/decalcifier for safranin O/fast green staining. Anti-TNF-α immunohistochemical analysis was applied to each knee joint. Briefly, 5-μm-thick sections of frozen tissue blocks were stained with mouse monoclonal anti-TNF-α antibody (1:1,000) and peroxidase-conjugated goat anti-mouse immunoglobulin G (H + L) secondary antibody (1:5,000) for further detection.

### Statistical Analysis

The statistical analyses were performed as the mean ± SD by one-way ANOVA with a value of *P < 0.05 being considered statistically significant.

## Results

### Preparation of PDAPEI

**Figure 1:**
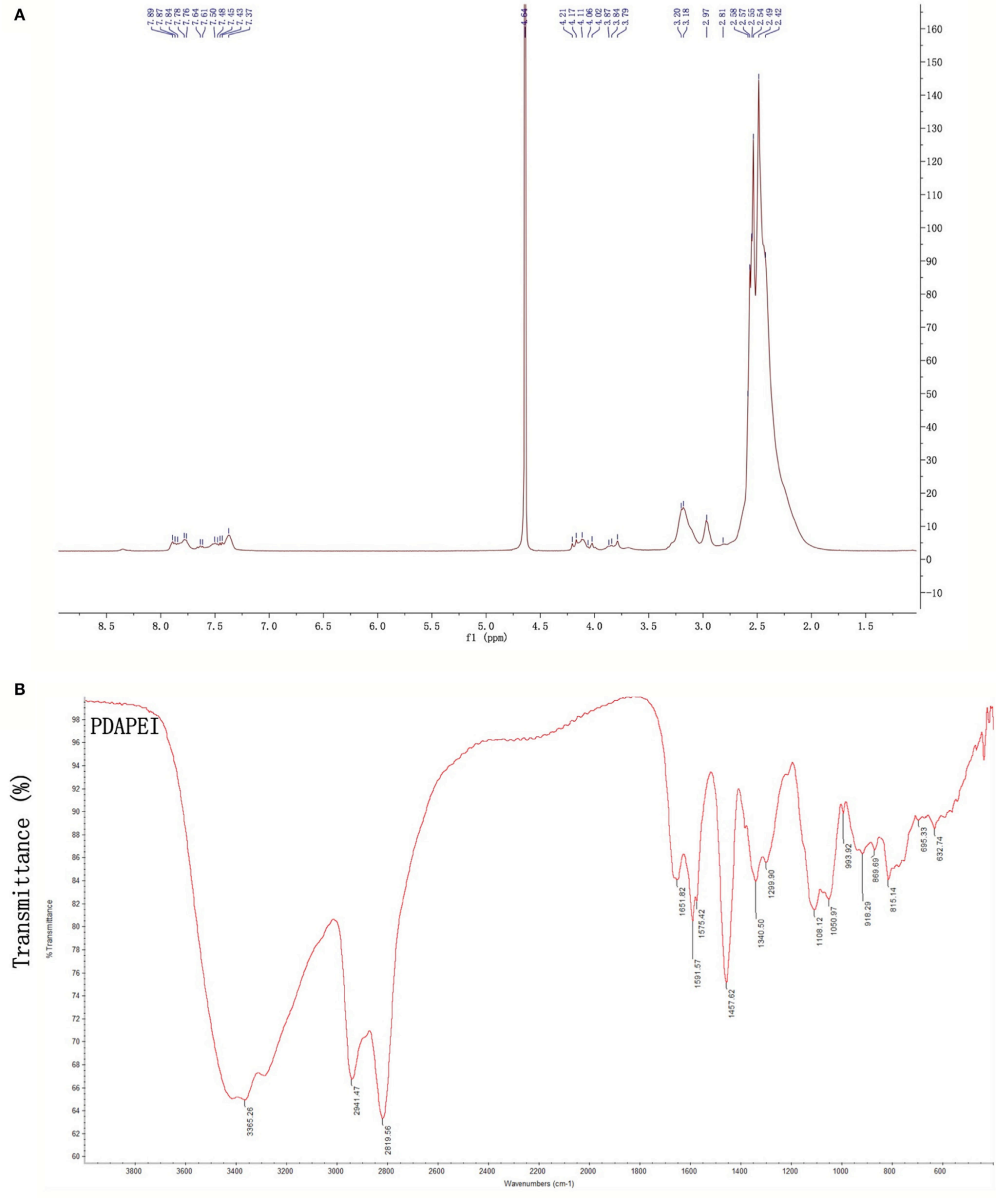
**The PDAPEI structure was confirmed using ^1^H NMR and FT-IR**. **(A)** NMR of PDAPEI. Chemical shifts at δ7.7 ppm of the ^1^H NMR spectrum are attributed to the protons of 2,6-pyridinedicarboxaldehyde. **(B)** IR of PDAPEI. This attribution is in agreement with the stretching vibration of the carbonyl group at 1,651 cm^−1^ in the FT-IR spectrum for 2,6-Pyridinedicarboxaldehyde carbonyls.

Proton nuclear magnetic resonance (^1^H NMR) and Fourier transform infrared spectroscopy (FT-IR) were used to confirm the structure of PDAPEI. The chemical shifts of ^1^H NMR and transmittance attribution in FT-IR were in agreement and as had been desired. The average molecular weight of PDAPEI was 21,000 Da, as measured by gel permeation chromatography (Figures 1A,B).

### Preparation and Characterization of the PDAPEI/pDNA Complex

**Figure 2:**
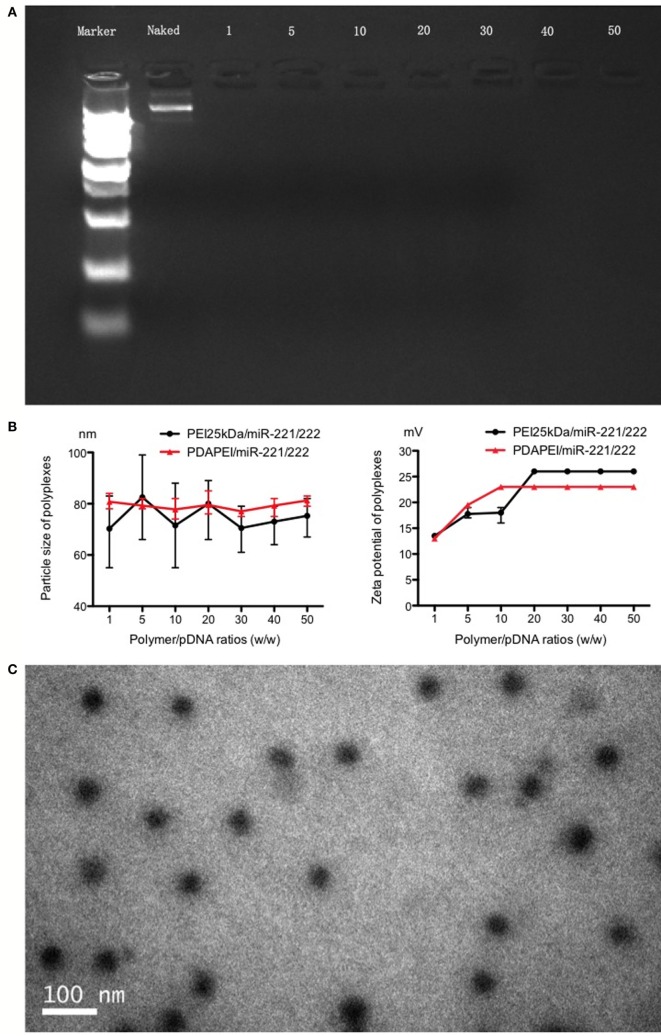
**Characteristics of PDAPEI/pDNA complexes**. **(A)** Gel electrophoresis indicated that PDAPEI condensed TNF-α shRNA completely at the w/w ratio of 1 or above (w/w = PDAPEI/TNF-α shRNA). **(B)** Particle size and zeta potential of polymer/DNA complexes (different w/w). **(C)** Morphology of PDAPEI/pDNA is observed by transmission electron microscopy (TCM).

The complex was successfully made per the above instructions. As shown in Figure 2, in the agarose gel electrophoresis assay, particle size and Zeta potential measurement and morphology analysis confirmed the formation and characterization of PDAPEI/pDNA complex. The gel electrophoresis indicated that PDAPEI condensed pDNA completely at a w/w ratio of 1 (Figure 2A). The particle size of PDAPEI/pDNA was stable at 80 nm, with a variety in w/w from 1 to 50, showed almost no difference compared with PEI 25 kDa/pDNA (Figure 2B left). PDAPEI/pDNA complex and PEI 25 kDa/pDNA (PH = 7.4) kept constant positive charges of ~22 mV with increased w/w ratio (from 10 to 40) (Figure 2B right). The Zeta potential of PDAPEI/pDNA complex (at w/w ratio of 20) was around 57 mV in acidic environment (pH = 5.4) and −9 mV in alkalinity environment (pH = 9.4), which decreased with increasing pH. The morphological analysis under TEM shoed the particle had a uniform diameter and spherical shape, consistent with the particle size measurement (Figure 2C).

#### *In Vitro* Cytotoxicity and Transfection Efficiency

**Figure 3:**
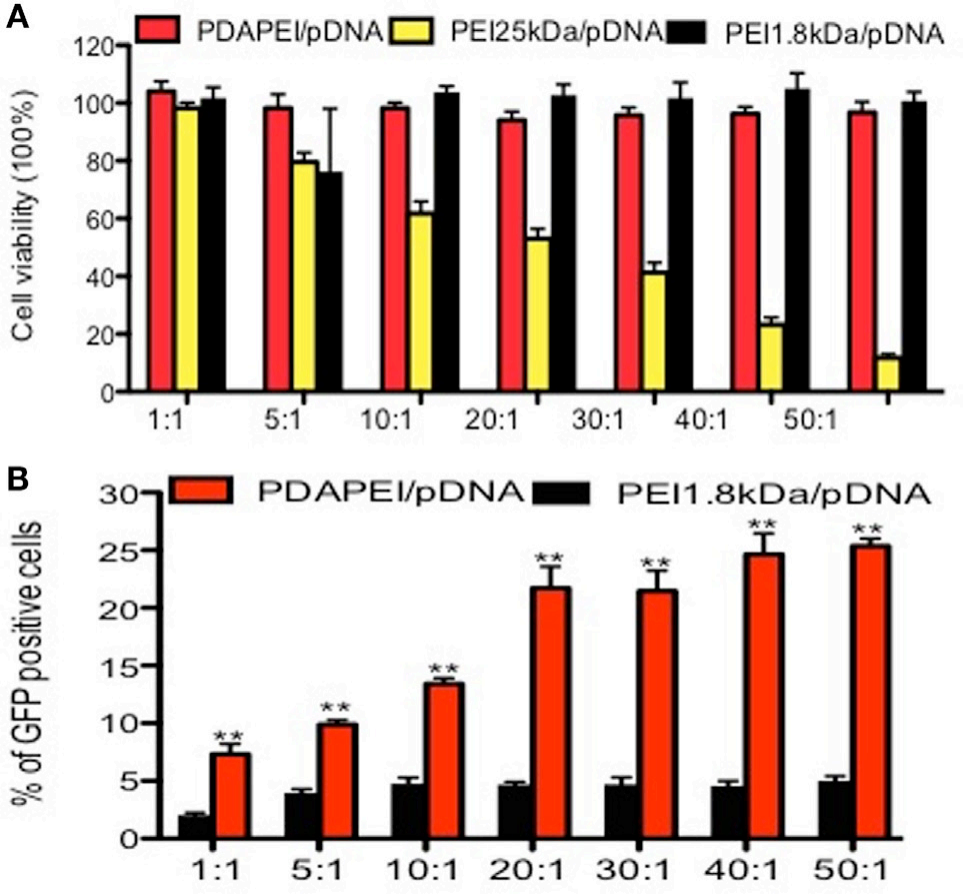
**Cytotoxicity and transfection efficiency *in vitro***. **(A)** Cytotoxicity of PDAPEI to RAW246.7 cells tested by CCK-8 assay (viability cells %) at different w/w. **(B)** Transfection efficiency of PDAPEI to RAW246.7 cells measured with flow cytometry (EGFP positive cells %) at different w/w (red: PDAPEI/pDNA, yellow: PEI 25 kDa/pDNA, black: PEI 1.8 kDa/pDNA). The experiments have been repeated for three times. (*p < 0.05, **p < 0.01).

The cytotoxicity of PDAPEI to RAW246.7 was determined by the CCK-8 assay and the results were shown in Figure 3A. Compared with PEI25 kDa/pDNA control group, PDAPEI/pDNA showed significantly lower cytotoxicity at different w/w ratios. With the w/w ratios increasing from 1 to 50, PDAPEI/pDNA complex was kept at a low cytotoxicity, with cell viability higher than 90%, there was no difference with the PEI 1.8 kDa/pDNA control group.

The GFP-positive RAW246.7 was observed with a fluorescence microscope. Almost no fluorescence was observed in the PEI 1.8 kDa/pDNA group and PBS control group, while obvious fluorescence was observed in the PDAPEI/pDNA and PEI 25 kDa/pDNA group. The flow cytometry results demonstrated that the PDAPEI/pDNA group showed a significantly higher GFP-protein level than PEI 1.8 kDa/pDNA at all w/w ratios. With the w/w ratio increasing from 1 to 50, the percentage of GFP-positive cells increased from 8 to 25%. Compared with PEI 25 kDa/pDNA (w/w ratio of 2), the PDAPEI/pDNA showed a similar percentage of GFP-positive cells (w/w ratio from 1 to 10) and a significantly higher percentage of GFP-positive cells (w/w ratio of 20–50) (Figure 3B).

#### Expression of TNF-α *In Vitro*

**Figure 4:**
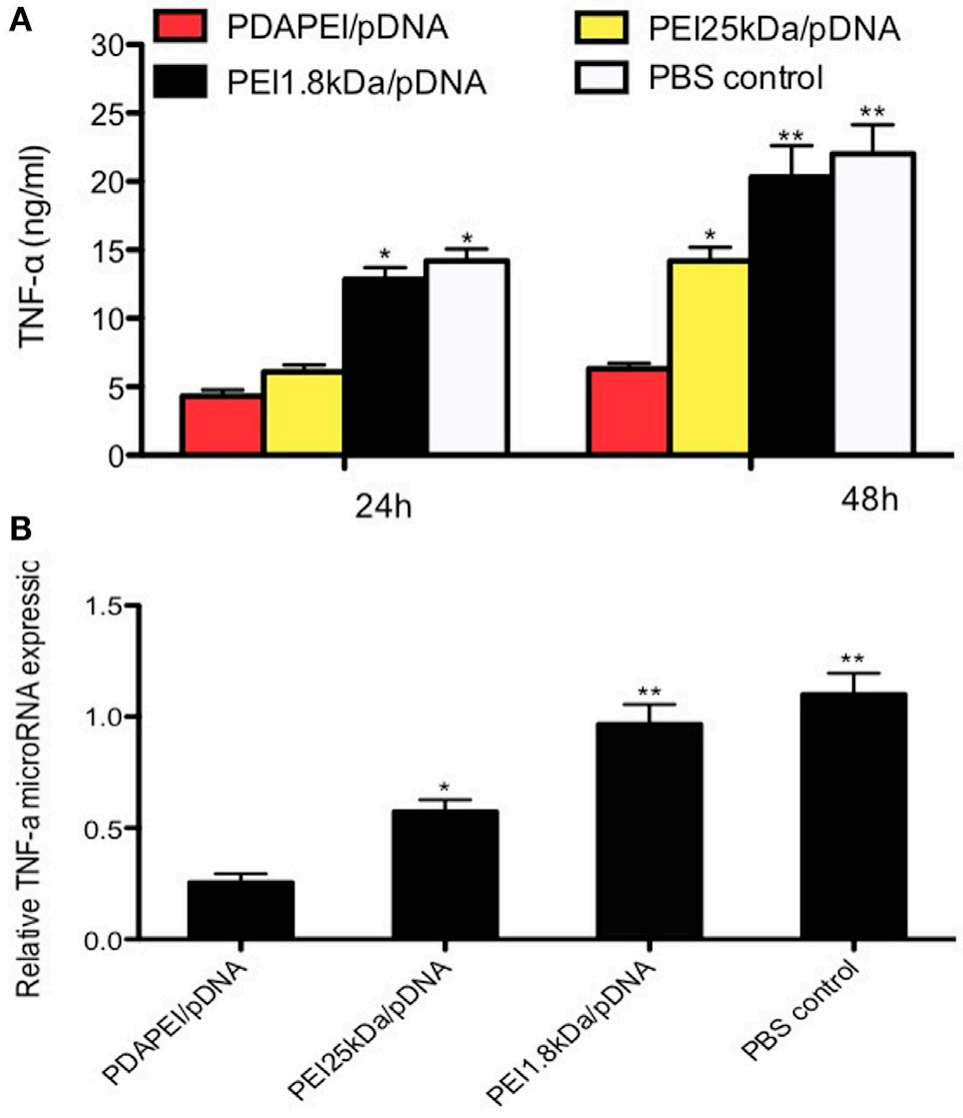
**Expression of TNF-α *in vitro***. **(A)** TNF-α expression of RAW246.7 cells determined by ELISA measurement 24/48 h after transfected with TNF-α shRNA by different polymers. **(B)** Relative levels of TNF-α mRNA expression was corrected by GAPDH. Data represent fold change of relative TNF-α expression normalized to GAPDH levels. (*p < 0.05, **p < 0.01).

The expression of TNF-α *in vitro* was measured by ELISA. As shown in Figure 4A, TNF-α in the supernatant was significantly reduced in the PDAPEI/pDNA group compared with other three groups after 24-h transfection. The concentrations of TNF-α were 22 ± 5.4 and 20.1 ± 6.5 ng/ml in the PBS control group and PEI 1.8 kDa/pDNA group, respectively, significantly higher than that of the PEI 25 kDa/pDNA group (14.2 ± 2.4 ng/ml, *p* < 0.05) and PDAPEI/pDNA group (6.1 ± 1.2 ng/ml, *p* < 0.01) 48 h after transfection. To further determine the *in vivo* silencing effect of TNF-α shRNA transfected by PDAPEI, real-time RT-PCR was utilized to measure the TNF-α microRNA expression. The mean levels of TNF-α microRNA expression in the PDAPEI/pDNA group were 23% of those in the PBS control group 48 h after transfection (Figure 4B).

#### Animal Model and Clinical Examination

**Figure 5:**
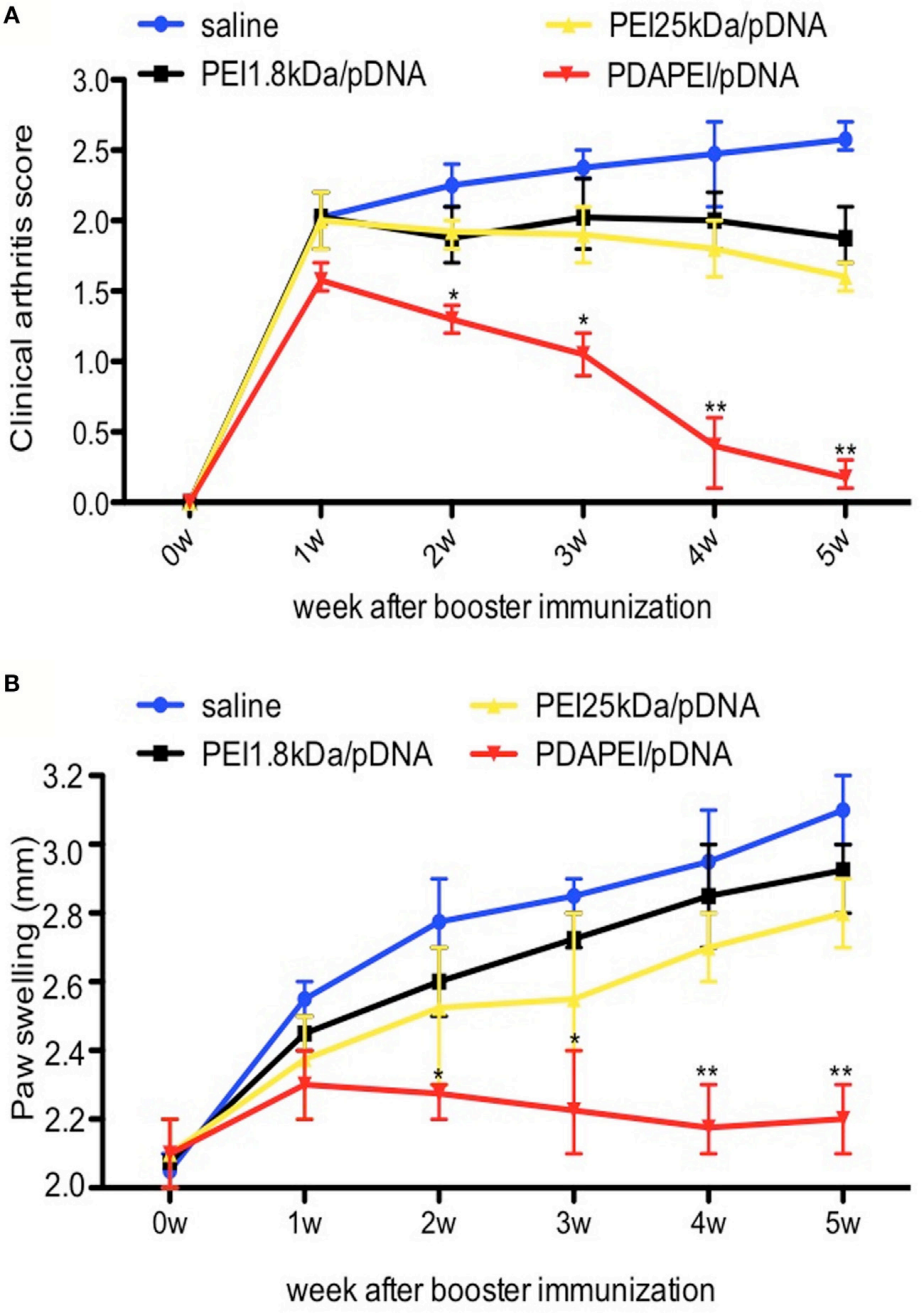
**Clinical scores of arthritis symptoms**. **(A)** Clinical arthritis score. **(B)** Paw swelling. Data were monitored every week after booster immunization, presented as mean ± SEM (*n* = 6 in each group, *p < 0.05, **p < 0.01).

The mouse CIA model was used to determine the effects of PDAPEI/pDNA on RA. To get 24 CIA mice, there were 21 mice without symptoms after booster immunization and these mice were eliminated from further study. The w/w ratio of PDAPEI/pDNA chosen was 20 according to previous *in vitro* results. No severe complications were observed throughout the experiment. As shown in Figure 5, mice treated with PDAPEI/pDNA exhibited significant reductions in the severity of CIA. During weeks 1–5 after booster immunization, the arthritis severity score decreased from 1.5 ± 0.2 to 0.2 ± 0.1, showing a significant difference when compared with saline group. The arthritis score for the saline control group was 2.5 ± 0.5 during the first week after booster immunization and maintained that score level throughout the experiment. The PEI 1.8 kDa/pDNA and PEI 25 kDA/pDNA groups lower scores than saline, but were not of significant difference (Figure 5A). We also examined edema by measuring the thickness of hind paw and found the results corresponded with the arthritic scores (Figure 5B).

### TNF-α Expression in Synovial Fluid of the Knees

**Figure 6:**
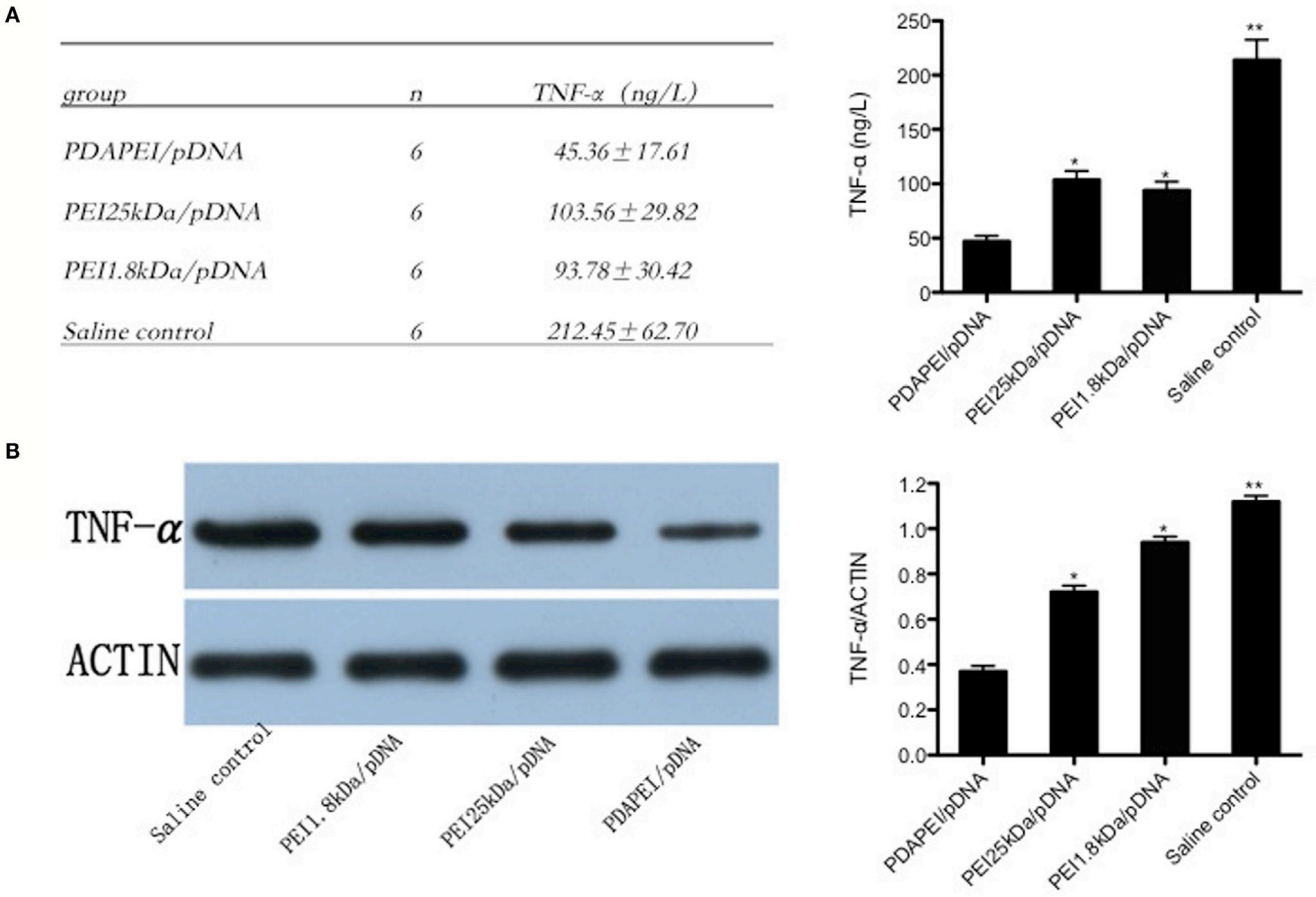
**TNF-α expression in synovial fluid of the knees**. **(A)** TNF-α levels in synovial fluid of the knees from four groups (PDAPEI/pDNA, PEI 25 kDa/pDNA, PEI 1.8 kDa/pDNA, and saline control group) were determined by enzyme-linked immunosorbent assay (ELISA). Values are the mean and SEM from six different mice. **(B)** Western-blot analysis of TNF-α levels in synovial fluid of the knees (*n* = 6 in each group, *p < 0.05, **p < 0.01).

Enough synovial fluid obtained from the knee in each group (0.1 ml at least) was used for ELISA and western-blot analysis. The level of TNF-α expression in synovial fluid of the knees was 212.45 ± 62.70 ng/L in the saline control group, and a substantial decrease in the production of TNF-α was found in the PDAPEI/pDNA group (45.36 ± 17.61 ng/L, *p* < 0.01). The levels of 11 in both the PEI 25 kDA/pDNA (103.56 ± 29.82 ng/L) and PEI 1.8 kDa/pDNA (93.78 ± 30.42 ng/L) groups were significantly higher than that in the PDAPEI/pDNA group, but lower than that in the saline control group (*p* < 0.05) (Figure 6A). The western-blot analysis corresponded with the ELISA results (Figure 6B).

### Histological Examination

**Figure 7:**
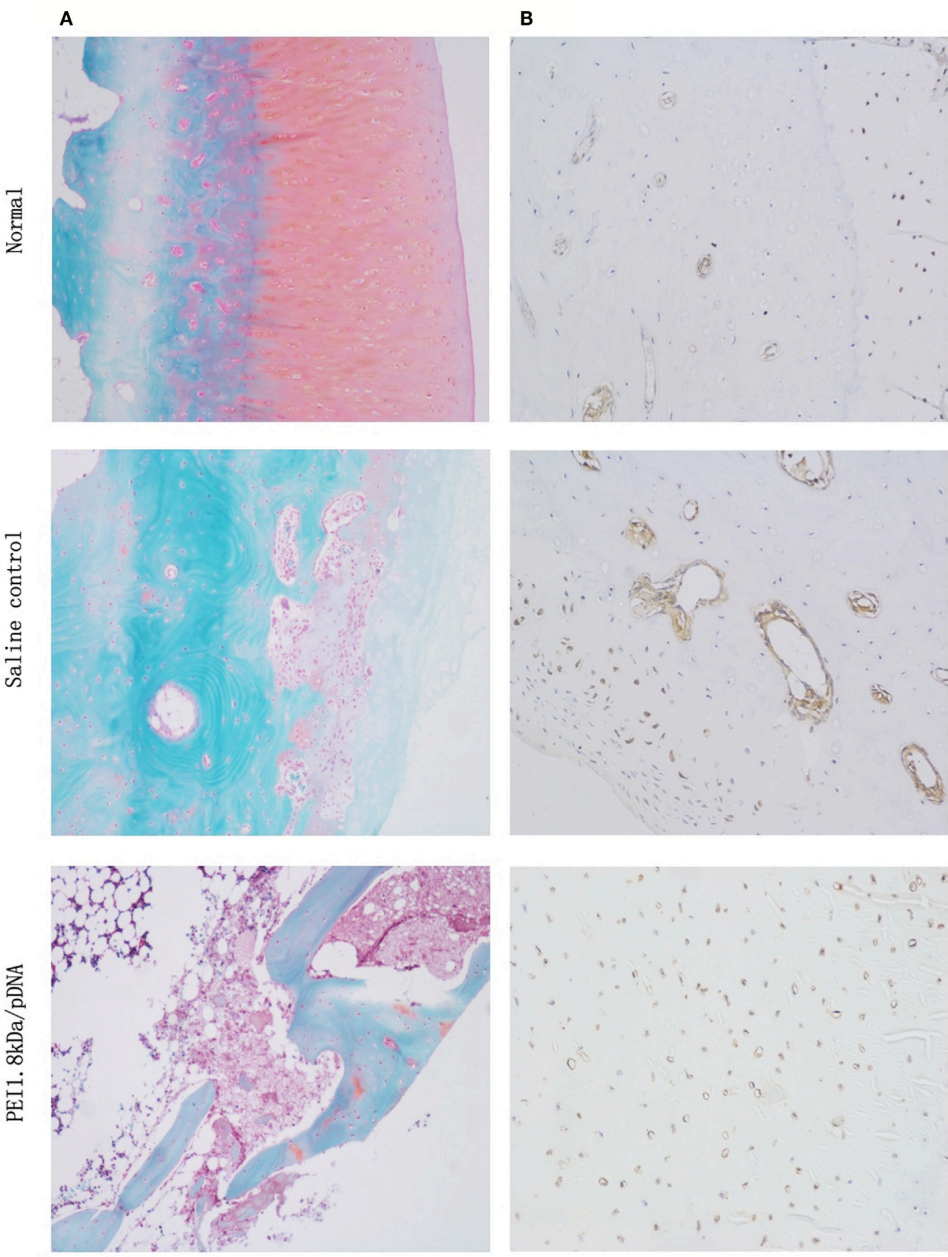

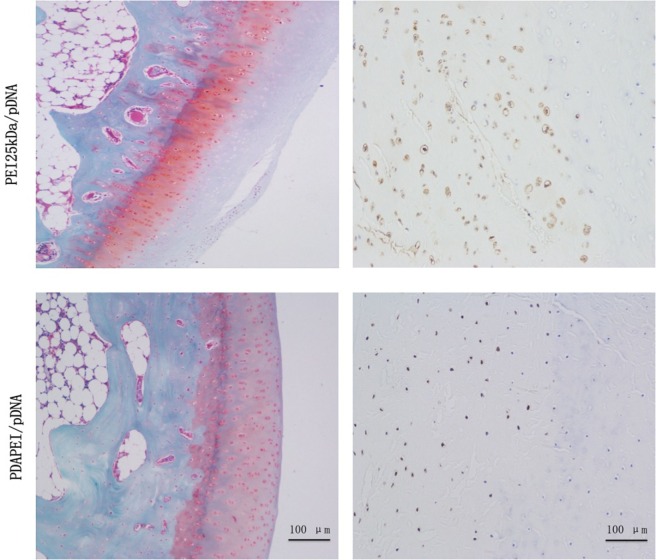
**Histological examination**. **(A)** Safranin O/fast green staining (red: cartilage, gray green: cytoplasm). **(B)** anti-TNF-α staining (brown: TNF-α) of 4 experimental group (PDAPEI/pDNA, PEI 25 kDa/pDNA, PEI 1.8 kDa/pDNA and saline control group) and normal knee joint. Scale bar = 100 μm.

Safranin O/fast green staining and anti-TNF-α immunohistochemical analysis were used for to examine the histology. As shown in Figure 7A, the histological cross-sections of knee joints revealed almost normal safranin O-stained (red) proteoglycans in the PDAPEI/pDNA group, with a smooth cartilage surface. Almost no proteoglycans were left in the saline control group. TNF-α immunohistochemistry staining showed that TNF-α was abundant in knee joint tissues taken from the saline control group. However, the signal for TNF-α staining in the PDAPEI/pDNA group was hardly visible, revealing a marked inhibition of TNF-α protein levels (Figure 7B).

## Discussion

The aim of this study was to develop an efficient and non-toxic delivery system for TNF-α shRNA in RA. RNA interference is the ability to exogenously introduce double-stranded RNA (dsRNA) molecules to inhibit the expression of specific gene homologs (13, 28). It has been utilized to effectively treat a variety of diseases, such as viral infections, cancer, and autoimmune disorders, by blocking gene expression in its target cells (29, 30). An optimal vector is needed to deliver the gene with high transfection efficiency and low cytotoxicity. In this study, PDAPEI condensed pDNA completely at a w/w ratio of 2, formed a homogeneous sphere with a particle size of 80 nm with a stable positive zeta potential of 22 mV. Particle size and zeta potential demonstrate the ability of DNA binding and affect cellular endocytosis and gene transfection efficiency (31). These characters made PDAPEI a perfect vector for gene transfer. We found a cell viability score of more than 90% and had higher transfection efficiency than with the PEI 25 kDa positive control. Different cationic polymers as gene carriers for Raw 264.7 cell have been investigated. Mannosylated chitosan-graft-PEI and degradable cationic monomers have shown great transfection ability, and the transfection efficiency is more than 40%, while the cell viability after transfection is lower than 80% (32–34). PDAPEI has the advantages of being non-toxic and possessing efficient transfection efficiency. One possible explanation is PDAPEI can metabolize itself into non-toxic PEI 1.8 kDa and 2,6-PDA under acidic environment conditions.

TNF-α plays a central role in the development and progression of RA and the main gene target for treatment (35). In our *in vitro* study, TNF-α shRNA was successfully transfected into macrophages and its expression level was significantly decreased. PDAPEI/pDNA had significantly better TNF-α inhibition results than the other three groups because of its high transfection efficiency and low cytotoxicity. TNF-α participates in cartilage/bone degradation and promotes other the secretion of other cytokines (36). Upon histological examination, anti-TNF-α immunohistochemical analysis indicated that PDAPEI/pDNA suppressed the expression of TNF-α. Furthermore, the PDAPEI/pDNA group yielded a better clinical examination result. These results demonstrate that PDAPEI/pDNA decreased the severity of arthritis in mice with CIA by inhibiting TNF-α expression.

Rheumatoid arthritis is a systemic autoimmune disease, characterized by non-organ-specific autoantibody production and chronic inflammation of the synovial tissues, leading to cartilage and bone destruction (37). Treatments, such as anti-TNF-α monoclonal antibodies, have been successfully developed but had limited application due to complications (38, 39). However, TNF-α shRNA is a powerful tool to specifically silence gene expression, and the repair results further proved its outcome in our experiment.

In conclusion, our results have shown that PDAPEI is an efficient delivery vector for TNF-α shRNA into macrophages. PDAPEI/pDNA can significantly inhibit TNF-α expression *in vitro* and proved to have an excellent efficacy in decreasing the severity of arthritis in mice with CIA. PDAPEI delivery of TNF-α shRNA has great potential in the treatment of RA.

## Author Contributions

JS and YC participated in its design, did tests, searched databases, extracted and assessed studies, and helped to draft the manuscript. SJ, KY, XL, and XZ participated in doing some tests. YO, CF, and WY participated in the conceptualization and design of data extraction and analysis, and wrote and revised the manuscript. All authors read and approved the final manuscript.

## Conflict of Interest Statement

The authors declare that the research was conducted in the absence of any commercial or financial relationships that could be construed as a potential conflict of interest. The reviewer SH and handling Editor declared their shared affiliation, and the handling Editor states that the process nevertheless met the standards of a fair and objective review.
